# Quality of Life Among Natural Menopausal Women and Early Surgical Menopausal Women: A Study from Greece

**DOI:** 10.3390/nursrep14040250

**Published:** 2024-11-11

**Authors:** Fotini Kavga, Anastasia Bothou, Christina Nanou, Giannoula Kyrkou, Victoria Vivilaki, Anna Deltsidou

**Affiliations:** 1 Rea Maternity Hospital, 17564 Athens, Greece; fot_ka@windowslive.com; 2 Department of Midwifery, University of West Attica, 12243 Athens, Greece; nanouxv@uniwa.gr (C.N.); ikirkou@uniwa.gr (G.K.); vvivilaki@uniwa.gr (V.V.); adeltsidou@uniwa.gr (A.D.)

**Keywords:** menopause, QOL, postmenopause, natural menopause, surgical menopause, nursing

## Abstract

Background/Objectives: The general health and well-being of middle-aged women have become a major public health issue worldwide. More than 80% of women experience physical or psychological symptoms during the transition to menopause. This study aims to compare the effect of menopause on quality of life (QOL) in two groups of women undergoing natural and surgical menopause. Methods: The sample consisted of 100 female patients from a Greek hospital in Athens, with an average age of 44.5 years, half of whom had natural menopause, while the remaining women had iatrogenic menopause after surgery for any reason other than malignancy. A questionnaire related to the QOL in menopause was used to collect the data. The scale used to evaluate the QOL of women is the Utian QOL Scale (UQOL), translated into Greek. Results: From the analysis of the data, it was found that there is no statistically significant difference between the QOL of women with natural and surgical menopause. Menopausal symptoms, psychosocial and sexual health, as well as the general health of the two groups, showed similar rates (OR: 63.7–66.6, *p* = 0.248). The only statistically significant difference found was in weight gain, with natural menopausal women having greater weight gain compared to surgically menopausal women (*p* = 0.041). Conclusions: Menopausal symptoms are associated with a decrease in women’s QOL. However, QOL is affected regardless of the type of menopause transition. This study was not registered.

## 1. Introduction

Quality of life (QOL) is defined by the World Health Organization as “individuals” perception of their position in life in relation to their goals, expectations, standards, and concerns as well as in the context of the culture and value systems in which they live [1]. The QOL is influenced by a person’s physical and mental well-being, degree of independence, social connections, personal beliefs, and interactions with prominent elements of their environment. Since the mid-20th century, QOL has been a point of interest for many researchers and clinicians in various health and physiological well-being issues. The menopause is not exempt from this, due to the increase in life expectancy, the importance of women’s health in terms of this period of life being as important as the reproductive period.

The term “menopause” refers to the natural end of a woman’s ability to conceive, which can also be artificially caused through bilateral oophorectomy, which may or may not involve the removal of the uterus and fallopian tubes [2]. Both types of menopause in women are characterized by low plasma levels, low concentrations of estradiol and progesterone in the brain, and a significant increase in follicle-stimulating hormone (FSH) levels [3].

This likely affects several brain neurotransmitter systems and some peripheral physiological processes, affecting women’s QOL [3,4]. Moreover, the long-term absence of steroid hormones is associated with physiological changes that predispose women to urogenital, cardiovascular, bone, and mood disorders [3]. While such changes occur gradually and require a long time to stabilize in natural menopause, they establish in a shorter time in surgical menopause, thus affecting the severity of symptoms compared to natural menopause [3,5,6,7].

However, in both cases, the typical symptoms associated with menopause significantly affect women’s QOL [8]. This study is an attempt to explore the differences in QOL between women with natural and surgical menopause. The novelty of this study was that this was the first study in Greece that approached this topic.

## 2. Materials and Methods

### 2.1. Design of the Study

The data in this study were primary, as they were collected by the researcher for the first time. Purposive sample of female patients of one hospital in Greece was applied as the sampling method. In this study, the method of data collection was carried out using a questionnaire in printed form, which was completed by patients who attended the hospital for several reasons (routine screening, follow-up, etc.), after giving informed consent. The necessary condition for the participants to be in natural or surgical menopause constitutes the inclusion criterion. On the other hand, in the case of surgical menopause, the main criterion of exclusion was that the surgery was performed because of a malignancy.

### 2.2. Ethical Issues

The study was conducted in accordance with the Declaration of Helsinki and approved by Rea Maternity Hospital in Athens, Greece, and the Midwifery Department of the University of West Attica with ethics approval number 1820/11-11-2023. The date of approval was 11 November 2023.

### 2.3. Research Tool

After searching the literature, no validated questionnaire was found to meet the needs of this study. Thus, a questionnaire was redesigned, but it was based on similar questions from other studies or literature [5,6,9,10,11] regarding sociodemographic data and medical history. The questionnaire was anonymous, and patients participated voluntarily. The questionnaire recorded the socio-demographic and anthropometric characteristics of the patients, the general health status of the patients, and the medical gynecological history of each patient. The Utian QOL Scale (UQOL) was used to assess patients’ QOL [12,13,14,15]. The Utian scale is a tool used to measure QOL in menopausal women; the reliability and validity of the instrument were assessed and published [12]. Every question on the UQOL had a five-point Likert-type score, and the question scores in each domain were added to create the domain scores. It is a self-administered psychometric instrument that includes 23 questions related to 4 distinct but interrelated dimensions of QOL (occupational, health, sexual and emotional). This scale is always administered with a questionnaire recording menopausal symptoms.

### 2.4. Statistical Analysis

The sample size was calculated by G-Power, version 3.1.9.7 (University of Düsseldorf, Düsseldorf, Germany) in order to have statistically significant results. The mean and standard deviation (SD), as well as the median and interquartile range, were used to describe the quantitative variables, after appropriate testing of the normality of the distribution through the Kolmogorov–Smirnov statistical test, and the absolute (N) and relative frequency (%) were used to describe the qualitative variables. To compare the distribution of quantitative variables between two categories, the *t*-test for two independent samples was used.

Pearson’s X^2^ test was used for the correlation between two categorical variables, or Fisher’s exact test in some cases, due to not fulfilling the conditions of Pearson’s X^2^ test. The independent factors linked to the variables under investigation were identified using stepwise linear regression analysis in conjunction with linear regression analysis, yielding coefficients of dependency (β) and standard errors (SE). All tests performed were two-sided, and statistical significance was set at the level of *p* ≤ 0.05. SPSS v. 24.0 statistical package was used for statistical analysis and presentation of results.

## 3. Results

### 3.1. Demographics

The present study included 100 women, half of whom had natural menopause (N = 50), while the remaining (N = 50) women underwent surgical menopause. The mean age of the participants was 44.5 years (SD = 2.3 years), and the time period since the onset of menopause was approximately more than one year. Forty-six percent of the women were married, and in terms of employment status, at least 8 out of 10 women (81%) reported working, while 18% were unemployed. Also, as shown in Table 1, there was a statistically significant difference in the age of participants according to their menopausal status, with women who had natural menopause being statistically significantly older (Mean = 46.0 years, SD = 1.6 years) compared to women who underwent surgical menopause (Mean = 43.0 years, SD = 1.8 years) (*p* < 0.001) [Table 1].

### 3.2. Anthropometric Characteristics

The participating women were, on average, 1.70 m tall and weighed 74.5 kg. Regarding their BMI, on average, it was equal to 27.2 kg/m^2^, 36% of the women were of normal body weight, 33% were overweight, and 30% of the women were obese. However, no statistically significant difference was found according to the menopausal status [Table 2].

### 3.3. Gynecological Characteristics

Eighty-one percent of the participants mentioned that the age of their first menstrual period was 12.2 years on average. Meanwhile, 67% of women reported that their menstrual periods were regular, while 50% of women reported having a uterus, cervix, and both ovaries. In addition, at least half of the women (56%) said they had breast examination. Finally, 22% of women had an abnormality on their Pap test, 31.3% had an abnormality on their mammogram, and 23.2% had an abnormality on their thyroid test. Furthermore, among women with natural menopause, those who have a uterus, those who have both ovaries, those who have a cervix, and those who had their breasts examined were statistically significantly higher than the percentage of women who have undergone surgical menopause. On the other hand, the proportion of women with regular menstrual periods was statistically significantly higher among women who underwent surgical menopause.

### 3.4. Health Profile

A total of 58% of participants experienced mood swings, 52% anxiety, 47% fatigue, and 40% of participants stated that their weight had increased. At the same time, at least 3 in 10 women said they had high blood pressure (hypertension) (38%), anemia (38%), muscle pain (35%), migraines (31%), arrhythmias (30%), and back pain (30%). Finally, there was a statistically significant difference in the percentage of women who gained weight, with this percentage being statistically significantly higher among women with natural menopause (50%), compared to the percentage of women who underwent surgical menopause (30%) (*p* = 0.041) [Table 3].

### 3.5. Menopausal Effect on Family Changes

The study found a statistically significant difference in the menopausal effect on family changes between the two participant groups, especially in the relationships between the family members and, in some cases, in the marital status. Specifically, women who had undergone surgical menopause had a statistically lower percentage of these changes than women who had undergone natural menopause [18 (36%) vs. 29 (58%), respectively] (*p* = 0.028).

### 3.6. Sexual Life

Although there are no statistically significant differences, it is important to highlight the high rates that the two groups have in terms of their sexual lives. More specifically, women who had naturally entered menopause showed concerns about their sexual life (85%), while 63% had lost interest in sexual activity. The same was true for the percentages of women who had surgery, with 64% having lost interest in sex, and 84% experiencing concerns about their sexual life. It was also found that women who have entered menopause either naturally or surgically have lost their ability to orgasm (88% vs. 92%) and during intercourse, they experience pain either from penile penetration, vaginal dryness, or vaginal pain.

### 3.7. Menopausal Symptoms

Both groups of women were affected by hot flashes whether their entry into menopause was natural (42%) or surgical (48%). This resulted in women in both groups having difficulty sleeping (38% vs. 36%), and lack of sleep created a constant fatigue that affected them in their daily lives (54% of women with natural menopause vs. 52% of women with surgical menopause). Moreover, women were found to be affected by estrogen deficiency, experiencing pain during intercourse (56%), lack of sexual interest (63% vs. 64%), and difficulty reaching orgasm (48% vs. 46%). Both groups of women show an alternation of emotions. A total of 57% of the patients showed several alternations, while 34% of them showed many alternations. Despite this high rate of emotional alternation, the women did not feel that their situation was bordering on depression.

### 3.8. QOL

Table 4 shows the participants’ scores on the UQOL according to their menopausal status. Higher scores indicate a better QOL. There was no statistically significant difference between the two groups [Table 4].

### 3.9. Effect of Various Characteristics on Total QOL Assessment Scores

Regarding the effect of different participant characteristics on their overall score on the QOL assessment tool (after the sequential variable removal inclusion procedure), the results of multivariate linear regression on the effect showed that

- Women who exercise almost every day scored 16.41 points higher than participants who do not exercise, indicating that they have a better QOL (*p* < 0.001).
- A 1 kg/m increase in BMI was associated with a 0.78 point decrease in their score, indicating that participants of higher weight have a significantly worse QOL (*p* = 0.002).
- Women who have not used other menopause treatment methods score 5.54 points lower, indicating that they have a significantly worse QOL (*p* = 0.018) [Table 5]. Participants’ scores on the dimensions “Quality of Professional Life” and “Emotional QOL” were not found to be significantly correlated with women’s characteristics.

## 4. Discussion

Menopause is a condition that causes physical and psychological changes in women’s QOL due to estrogen deficiency [16,17]. Furthermore, surgical menopause causes a sudden drop in estrogen levels.

In contrast, a naturally menopausal woman goes through a phase of fluctuating hormone levels, and although the majority of these women report troublesome symptoms when asked, only some of them are related to the hormonal changes of the menopause transition. Evidence from other studies supports that surgical menopause, compared to natural menopause, is associated with more severe psychological and physical symptoms [3,5,6,7,18].

However, our study found that the rates of menopause symptoms were significantly higher in both women who entered menopause naturally and women who underwent surgery. Our findings suggest that women, regardless of the type of menopause, suffered from severely different menopausal symptoms such as hot flushes, musculoskeletal and sweating symptoms, as well as depressed mood, anxiety, and sleep problems. Interestingly, despite that there is no statistical significance, our study indicated that in both groups, there was a high percentage of concerns about sexual life and lost interest in sexual activity. This result may be due to the significant decrease in estrogens that had an effect on vaginal dryness and painful intercourse (dyspareunia).

It is worth mentioning that urogenital symptoms including sexual problems, bladder problems, and vaginal dryness were less common in both groups. The individual and total UQOL scores were also low for the urogenital system, particularly more in women with surgical menopause, but the difference was not found to be statistically significant. This finding was also confirmed by the data in the study by Bhattacharya and Jha in 2010 [5].

The only statistically significant difference found in our study was in weight gain, with women with natural menopause having a higher rate compared to women who underwent surgical menopause. This result is similar to other studies, which showed that overweight women have lower self-esteem and sometimes more health problems, affecting their QOL [19,20,21,22,23].

In a multicentric cohort study called the Study of Women’s Health Across the Nation (SWAN) [23], it was found that women who gained weight after menopause experienced more vasomotor symptoms or hot flashes, and consequently had lower QOL than those whose weight was unchanged.

In conclusion, postmenopausal women’s QOL, which is impacted by a variety of social and personal factors, is often impacted by menopause and accompanying symptoms. Therefore, appropriate interventions through public health policy aimed at mitigating the symptoms of menopause and maintaining the QOL. A limitation of the present study is that the results cannot be generalized, and larger studies are needed to confirm them.

## 5. Conclusions

Menopausal symptoms are associated with a decrease in women’s QOL. However, QOL is affected regardless of the type of menopause (natural menopause or surgical menopause). The present study suggests that, in the study population, the type of menopausal entry does not seem to show statistically significant differences in the occurrence of symptoms and QOL of women. Menopausal symptoms, psychosocial and sexual health, as well as the general health of the two groups, showed similar rates. The only statistically significant difference found was in weight gain, with women with natural menopause having a higher rate compared to women who underwent surgical menopause. Interestingly, despite that there is no statistical significance, our study indicated that in both groups, there was a high percentage of concerns about sexual life and lost interest in sexual activity. However, further studies in larger populations are needed to clarify whether surgical menopause may affect women’s QOL to a greater extent than natural menopause.

## Figures and Tables

**Table 1:** Participant demographics.

		Total (N = 100)	Menopause Status		P-Pearson X^2^ Test
			Natural (N = 50)	Surgical (N = 50)	
		N (%)	N (%)	N (%)	
Age (Years) [Mean (SD)]		44.5 (2.3)	46.0 (1.6)	43.0 (1.8)	<0.001 ^1^
Marital status	Unmarried	16 (16.0)	7 (14.0)	9 (18.0)	0.715
	Married	46 (46.0)	21 (42.0)	25 (50.0)	
	Divorced	22 (22.0)	12 (24.0)	10 (20.0)	
	Widow	4 (4.0)	2 (4.0)	2 (4.0)	
	In symbiosis	12 (12.0)	8 (16.0)	4 (8.0)	
Working status	Unemployed	18 (18.0)	10 (20.0)	8 (16.0)	0.539
	Employee	81 (81.0)	40 (80.0)	41 (82.0)	
	Retired	1 (1.0)	0 (0.0)	1 (2.0)	

^1^ *p*-value was obtained from the Independent samples *t*-test.

**Table 2:** Anthropometric characteristics among the women participating in the study.

	Total (N = 100)	Menopause Status		P-Independent Samples *t*-Test
		Natural (N = 50)	Surgical (N = 50)	
	N (%)	N (%)	N (%)	
Height (in meters) [Mean SD]	1.7 (0.0)	1.7 (0.1)	1.7 (0.0)	0.817
Weight (in kilograms) [Mean SD]	74.5 (13.7)	75.5 (15.2)	73.4 (12.3)	0.452
BMI (in kg/m^2^) [Mean SD]	27.2 (4.5)	27.6 (5.1)	26.8 (3.9)	0.355
Underweight	1 (1.0)	1 (2.0)	0 (0.0)	0.298
Normal body weight	36 (36.0)	18 (36.0)	18 (36.0)	
Overweight	33 (33.0)	13 (26.0)	20 (40.0)	
Obese	30 (30.0)	18 (36.0)	12 (24.0)	

**Table 3:** Health status of the women who participated in the study.

		Total	Menopause		P-Pearson X^2^ Test
			Natural	Surgical	
		Ν (%)	Ν (%)	Ν (%)	
Migraines	No	69 (69.0)	37 (74.0)	32 (64.0)	0.280
	Yes	31 (31.0)	13 (26.0)	18 (36.0)	
Fatigue	No	53 (53.0)	22 (44.0)	31 (62.0)	0.071
	Yes	47 (47.0)	28 (56.0)	19 (38.0)	
Blood pressure	No	62 (62.0)	31 (62.0)	31 (62.0)	>0.999
	Yes	38 (38.0)	19 (38.0)	19 (38.0)	
Diarrhea	No	76 (76.0)	37 (74.0)	39 (78.0)	0.640
	Yes	24 (24.0)	13 (26.0)	11 (22.0)	
Sleepiness	No	72 (72.0)	36 (72.0)	36 (72.0)	>0.999
	Yes	28 (28.0)	14 (28.0)	14 (28.0)	
Constipation	No	91 (91.0)	45 (90.0)	46 (92.0)	0.727
	Yes	9 (9.0)	5 (10.0)	4 (8.0)	
Dizziness	No	81 (81.0)	41 (82.0)	40 (80.0)	0.799
	Yes	19 (19.0)	9 (18.0)	10 (20.0)	
Arrhythmias	No	70 (70.0)	35 (70.0)	35 (70.0)	>0.999
	Yes	30 (30.0)	15 (30.0)	15 (30.0)	
Mood swings	No	42 (42.0)	22 (44.0)	20 (40.0)	0.685
	Yes	58 (58.0)	28 (56.0)	30 (60.0)	
Muscle pains	No	65 (65.0)	35 (70.0)	30 (60.0)	0.95
	Yes	35 (35.0)	15 (30.0)	20 (40.0)	
Suicidal tendencies	No	99 (99.0)	49 (98.0)	50 (100.0)	0.315
	Yes	1 (1.0)	1 (2.0)	0 (0.0)	
Breast pains	No	86 (86.0)	42 (84.0)	44 (88.0)	0.564
	Yes	14 (14.0)	8 (16.0)	6 (12.0)	
Back pains	No	70 (70.0)	34 (68.0)	36 (72.0)	0.663
	Yes	30 (30.0)	16 (32.0)	14 (28.0)	
Hair loss	No	96 (96.0)	47 (94.0)	49 (98.0)	0.307
	Yes	4 (4.0)	3 (6.0)	1 (2.0)	
Varicose veins	No	89 (89.0)	44 (88.0)	45 (90.0)	0.749
	Yes	11 (11.0)	6 (12.0)	5 (10.0)	
Incontinence	No	72 (72.0)	32 (64.0)	40 (80.0)	0.075
	Yes	28 (28.0)	18 (36.0)	10 (20.0)	
Weight loss	No	86 (86.0)	43 (86.0)	43 (86.0)	>0.999
	Yes	14 (14.0)	7 (14.0)	7 (14.0)	
Indigestion	No	84 (84.0)	45 (90.0)	39 (78.0)	0.102
	Yes	16 (16.0)	5 (10.0)	11 (22.0)	
Depression	No	81 (81.0)	41 (82.0)	40 (80.0)	0.799
	Yes	19 (19.0)	9 (18.0)	10 (20.0)	
Nausea and vomiting	No	86 (86.0)	45 (90.0)	41 (82.0)	0.249
	Yes	14 (14.0)	5 (10.0)	9 (18.0)	
Anxiety	No	48 (48.0)	28 (56.0)	20 (40.0)	0.109
	Yes	52 (52.0)	22 (44.0)	30 (60.0)	
Weight gain	No	60 (60.0)	25 (50.0)	35 (70.0)	0.041
	Yes	40 (40.0)	25 (50.0)	15 (30.0)	

**Table 4:** QOL Scale (UQOL) score.

	Menopause				P-Independent Samples *t*-Test
	Natural		Surgical		
	Mean Value	SD	Mean Value	SD	
Occupational	22.6	5.1	23.0	5.2	0.727
Health	20.0	5.9	21.5	5.2	0.181
Emotional	14.9	3.6	16.2	4.4	0.120
Sexual	6.2	2.7	6.0	2.8	0.715
Total	63.7	11.6	66.6	13.4	0.248

**Table 5:** Correlation between exercise frequency and Overall QOL.

Dependent Variable: “Overall QOL”	Dependence Coefficient (β)	Standard Error (SE)	*p*-Value
How often do you exercise?			
Almost every day	16.41	3.63	<0.001
At least 3 times/week	6.09	3.11	0.050
Occasionally	3.13	3.48	0.369
Rarely	−2.29	3.85	0.552
BMI (per 1 kg/m^2^ increase)	−0.78	0.25	0.002
Have you used other menopause treatment methods?			
Νο	−5.54	2.33	0.018

## Data Availability

The data that support the findings of this study are available from the corresponding author upon reasonable request.

## Public Involvement Statement

There was no public involvement in any aspect of this research.

## Guidelines and Standards Statement

This manuscript was drafted against the Strengthening the Reporting of Observational studies (STROBE).
